# A high-fat diet induces a microbiota-dependent increase in stem cell activity in the *Drosophila* intestine

**DOI:** 10.1371/journal.pgen.1008789

**Published:** 2020-05-26

**Authors:** Jakob von Frieling, Muhammed Naeem Faisal, Femke Sporn, Roxana Pfefferkorn, Stella Solveig Nolte, Felix Sommer, Philip Rosenstiel, Thomas Roeder

**Affiliations:** 1 Zoological Institute, Department of Molecular Physiology, Kiel University, Kiel, Germany; 2 IKMB, UKSH, Kiel University, Kiel, Germany; 3 German Center for Lung Research, Airway Research Center North, Kiel, Germany; German Cancer Research Center (DKFZ), GERMANY

## Abstract

Over-consumption of high-fat diets (HFDs) is associated with several pathologies. Although the intestine is the organ that comes into direct contact with all diet components, the impact of HFD has mostly been studied in organs that are linked to obesity and obesity related disorders. We used *Drosophila* as a simple model to disentangle the effects of a HFD on the intestinal structure and physiology from the plethora of other effects caused by this nutritional intervention. Here, we show that a HFD, composed of triglycerides with saturated fatty acids, triggers activation of intestinal stem cells in the *Drosophila* midgut. This stem cell activation was transient and dependent on the presence of an intestinal microbiota, as it was completely absent in germ free animals. Moreover, major components of the signal transduction pathway have been elucidated. Here, JNK (basket) in enterocytes was necessary to trigger synthesis of the cytokine upd3 in these cells. This ligand in turn activated the JAK/STAT pathway in intestinal stem cells. Chronic subjection to a HFD markedly altered both the microbiota composition and the bacterial load. Although HFD-induced stem cell activity was transient, long-lasting changes to the cellular composition, including a substantial increase in the number of enteroendocrine cells, were observed. Taken together, a HFD enhances stem cell activity in the *Drosophila* gut and this effect is completely reliant on the indigenous microbiota and also dependent on JNK signaling within intestinal enterocytes.

## Author summary

High-fat diets have been associated with a plethora of morbidities. The major research focus has been on its effects on obesity related disorders, mostly omitting the intestine, although it is the organ that makes the first contact with all diet components. Here, we aimed to understand the effects of HFD on the intestine itself. Using *Drosophila* as a model, we showed that a HFD and more specifically, trigylcerides with saturated fatty acids, induced a transient activation of intestinal stem cells. This response completely depended on the presence of an intestinal microbiota, as in germ free flies this reaction was completely abolished. Mechanistically, we found that HFD induces JNK signaling in enterocytes, which triggers production of the cytokine *upd3*. This ligand of the JAK/STAT pathway, in turn activates STAT signaling in intestinal stem cells, leading to their activation. All these components of the JNK- and JAK/STAT-pathways are necessary for a HFD to lead to increased stem cell production. Moreover, HFD changed both, composition and abundance of the microbiota. As fecal transfer experiments failed to recapitulate the HFD phenotype, we assume that the increased bacterial load is the major cause for the HFD triggered stem cell activation in the intestine.

## Introduction

High caloric intake and especially high-fat diets (HFDs) are major causes of the epidemic increases in obesity-associated diseases [1]. In addition to metabolically relevant organs, the intestines are particularly susceptible to the effects of HFDs because they are in direct contact with constituents of the diet. Consequently, nutritional interventions directly impact intestinal structure and functionality [2]. Diet-dependent plasticity in the size of the intestines has been reported [3]. Specifically, intestinal size decreases in response to dietary restriction [4], but increases upon re-feeding after a period of starvation [5]. Structural changes in response to HFDs are observed at different levels, ranging from subcellular structures in enterocytes [6] to the cellular composition of the intestinal epithelium [7]. The effects of HFDs on the intestinal structure involve alteration of the activity of intestinal stem cells (ISCs). In mammals, HFDs directly enhance the activity of ISCs, leading to increased villi lengths in the small intestines via a mechanism involving ß-catenin signaling [8]. This HFD-induced activation of ISCs appears to be directly caused by the lipid content of food [3, 9]. A recent study showed that food with high lipid contents induces very robust PPAR-δ activation in ISCs and thereby increases the number of mitotically active cells in the intestines [9, 10]. Moreover, this response increases the tumorigenicity of intestinal progenitors [2, 9]. This observation correlates with epidemiological studies showing that different types of diets affect the risk of developing intestinal cancers [11, 12]. Specifically, HFDs increase the prevalence of colon cancers [13, 14]. In this context, deregulated stem cell activities appear to be directly associated with alterations in intestinal JAK/STAT signaling [15].

Although the major effects of HFDs on the stemness and tumorigenicity of ISCs seem to be directly mediated by exposure of intestinal epithelial cells to fat components [9], secondary effects are also highly relevant to induction of the complex phenotype that results from chronic consumption of a HFD. One important link between HFDs and disease development is the intestinal microbiota [16–18]. High-fat dietary supplementation alters the abundance, composition, and physiological performance of the microbiota [19–21]. In flies, HFDs increase the microbial abundance in the intestines [22]. Associations between an altered or dysbiotic microbiota composition and metabolic diseases have been repeatedly reported [23, 24]. Furthermore, microbiota transfer experiments revealed that altered microbiota compositions play a causative role in the development of metabolic disorders [25, 26]. In *Drosophila*, a link between a dysbiotic microbiota and the activity of ISCs was reported [27]. In this context, age-associated increases in the abundance of a particular microbial colonizer and the infection of specific pathogens trigger induction of stem cell activities [28, 29].

Despite this large body of informative studies, knowledge of the mechanisms by which HFDs regulate the activity of ISCs is not comprehensive. Here, we used the fruit fly *Drosophila* as a model and showed that a HFD induces a transient increase in stem cell activity via JNK-dependent activation of cytokine expression in enterocytes. This effect is dependent on the microbiota. Thus, we speculate that HFDs elicit physiological effects not only directly via activating stem cells through exposure to different fat components, but also indirectly via altering the microbiota, especially the bacterial abundance in the intestines.

## Results

**Fig 1:**
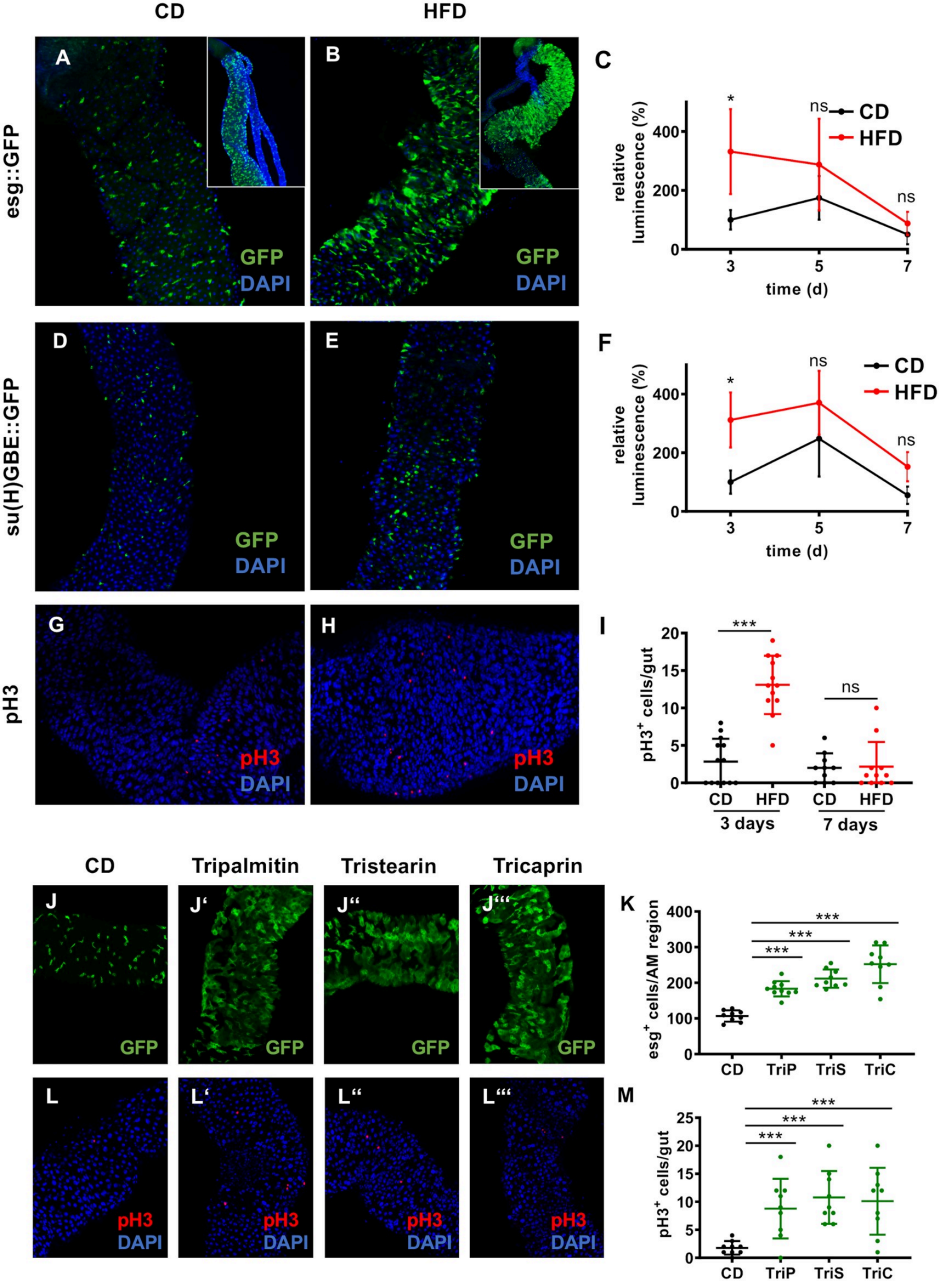
High-fat dieting leads to hyperproliferation of ISCs in the Drosophila intestine. (A–B) Anterior midgut region of flies with GFP-labeled ISCs and EBs (esg-Gal4::UAS-GFP) and fed a CD (A) or a HFD for 3 days (B). The insert in A shows a region of the posterior midgut from control animals and the insert in B shows a corresponding region of HFD treated animals. (C) Luciferase signals of esg^+^ cells ectopically expressing luciferase (esg-Gal4::UAS-luciferase). Intestines were dissected from flies fed a CD or HFD for 3, 5, and 7 days (n = 9–14). (D–E) Pattern of GFP expressed under the control of the enteroblast-specific su(H)GBE driver (su(H)GBE-Gal4::UAS-mCD8-GFP) in intestines of flies fed a CD (D) or HFD (E). (F) Quantification of luciferase signals in su(H)GBE^+^ cells of flies fed a CD or HFD (n = 9–11). (G–H) Representative images of anti-pH3 staining in the intestine of flies fed a CD (G) or HFD (H). (I) Quantification of the number of pH3^+^ cells in whole intestines at two different time points, 3 days and 7 days after start of HFD (n = 11–13). (J-J”‘) Representative images of the anterior midgut region of esg-Gal4::UAS-GFP flies fed a CD or a CD supplemented with 20% tripalmitin, tristearin or tricaprin. (K) Quantification of esg^+^ cells in the anterior midgut region of flies fed a CD or a tripalmitin, tristearin or tricaprin supplemented diet (n = 9–10). (L-L”‘) Representative images of intestines of flies stained with an anti-pH3 antibody after feeding a CD or a tripalmitin-, tristearin-, or tricaprin-supplemented diet. (M) Quantitative analysis of the number of pH3^+^ cells in whole intestines upon feeding a triglyceride-based diet in comparison to control dieting flies (n = 9). CD = control diet, HFD = high-fat diet, ISC = intestinal stem cell, EB = enteroblast, AM = anterior midgut, TriP = tripalmitin, TriS = tristearin, TriC = tricaprin. *p<0.05, ***p<0.001.

We used the fruit fly *Drosophila melanogaster* as a model to study the effects of a HFD on the structural and physiological characteristics of the intestines. Furthermore, we analyzed the contribution of the microbiota to these alterations. HFD feeding triggered a burst of stem cell activity in the midgut (Fig 1). We used flies that expressed GFP under the control of the escargot (esg) driver, meaning expression was restricted to ISCs and their direct descendants, i.e., enteroblasts (EBs). This analysis revealed that in comparison with flies fed a control diet (CD) (Fig 1A), short durations of HFD feeding induced substantial expansion of these cell types (Fig 1B). This reaction was seen throughout the entire midgut. A focus of our analysis was on the anterior midgut (AM), because this midgut region showed a low background of esg^+^ cells in control animals (Fig 1A). The inserts (Fig 1A and 1B) showed that the response was very similar in the posterior midgut (PM). Quantitative analysis of these cells was performed using flies that expressed luciferase in the same spatial pattern (Fig 1C). For this, complete midguts comprising AM and PM were used. Luminescence increased upon very short durations of HFD feeding, but it decreased over time (Fig 1C).

A similar increase in cell number upon HFD feeding was observed when only EBs were analyzed using flies that expressed GFP under the control of the su(H)GBE-Gal4 driver (Fig 1D–1F). After 3 days, more EBs were detected in flies fed a HFD (Fig 1E) than in flies fed a CD (Fig 1D). Similar results were obtained by quantitative analysis of luciferase expression (Fig 1F). As observed with esg-specific signals (i.e., ISCs plus EBs), luminescence remained elevated after 7 days of HFD feeding. The effects of different diets on ISC activity were directly evaluated by counting the number of phospho-H3 (pH3)^+^ cells in the gut. The number of pH3^+^ cells was increased after 3 days of HFD feeding (Fig 1H) in comparison with flies fed a CD (Fig 1G), which was further supported by a quantitative analysis of the numbers of pH3^+^ cells (Fig 1I). This effect on pH3^+^ cells was not seen after 7 days of HFD (Fig 1I). Palm fat is known to contain high concentrations of triglycerides with saturated fatty acids. To evaluate if these major constituents of palm fat can trigger the same reaction, we have used synthetic triglycerides that carry identical saturated fatty acids as substituents. We have tested glyceryl-tristearate (C18), glyceryl-tripalmitate (C16), and glyceryl-tricaprylate (C8) at concentrations of 20%, which corresponds to the concentration at which the palm fat was analyzed. In these experiments we observed a substantial expansion of esg^+^ cells both, in the anterior and posterior midgut (Fig 1J and 1K). Counting the numbers of esg^+^ cells in defined regions of the anterior midgut revealed statistically significant 2–2,5 fold increases in cell numbers depending on the triglyceride in use. Moreover, we quantified the numbers of pH3^+^ cells in these gut regions and also found significantly increased numbers of this cell population (Fig 1L and 1M).

**Fig 2:**
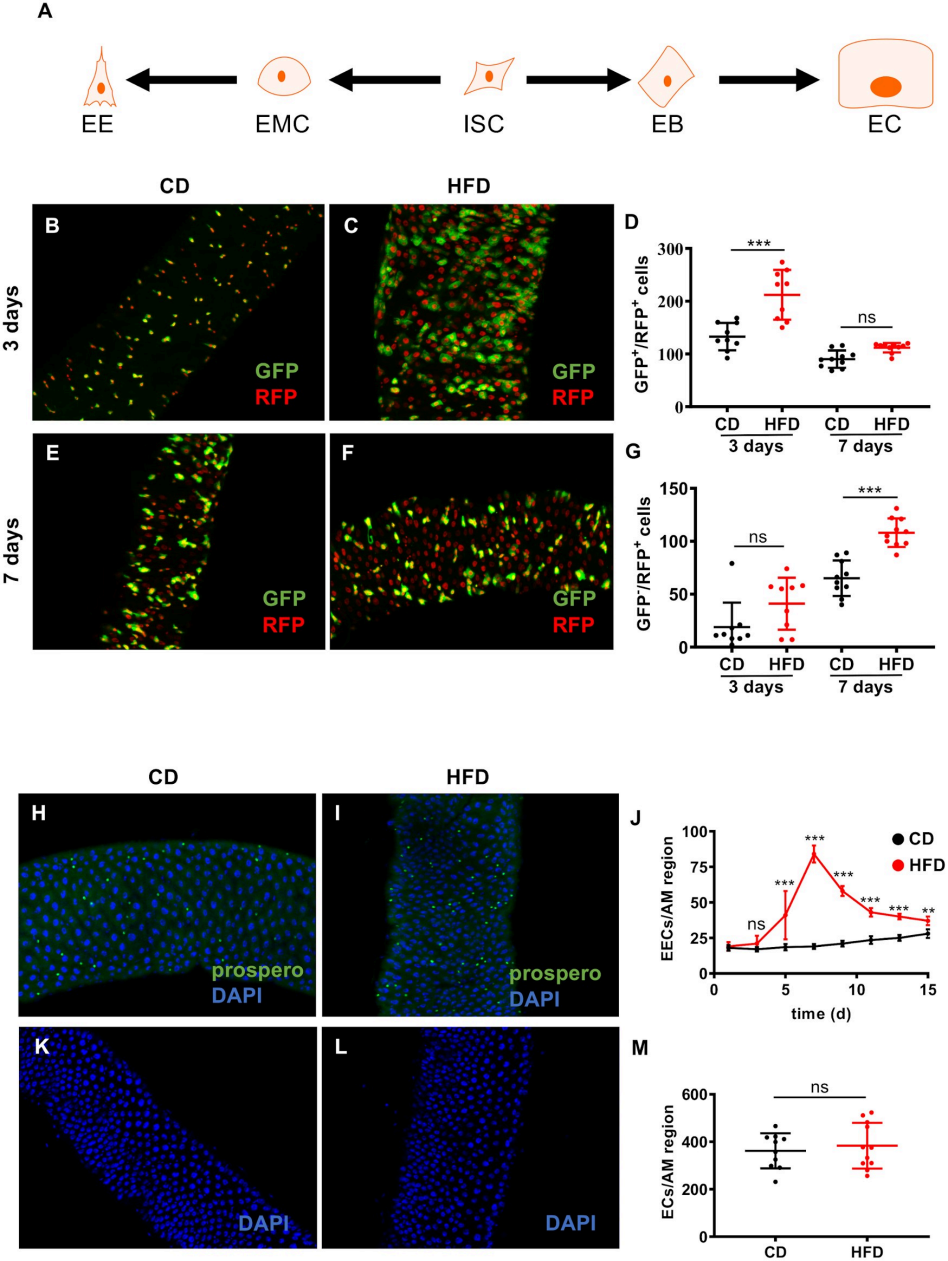
High-fat dieting affects the intestinal cellular composition. (A) Scheme of the generation of various cell types in the Drosophila midgut. ReDMM analysis of intestine from flies fed a control or a HFD (B-G). The ReDMM tracing method was combined with an esg-Gal4 driver. Therefore, ISCs and EBs are marked with mCD8::GFP and H2B::RFP, whereas renewed ECs and EECs are exclusively marked by H2B::RFP. Representative images of the anterior midgut region of ReDDM flies fed a CD (B) or a HFD (C) for 3 days. (D) Quantitative analysis of the number of GFP^+^ and RFP^+^ cells in the anterior midgut of ReDMM flies fed a HFD for 3 days and 7 days in comparison to control dieting flies (n = 10). (E, F) Representative images of the anterior midgut region of ReDMM flies fed a CD (E) or a HFD (F) for 7 days. (G) Quantitative analysis of GFP^**-**^ and RFP^+^ cells in the anterior midgut region of ReDMM flies fed a CD or HFD for 3 days and 7 days (n = 10). (H, I) Labeling of enteroendocrine (EECs) cells with the anti-prospero antibody in control intestines (H), and in HFD treated ones (I). (J) Number of EECs cells in the anterior midgut region of the intestines over 15 days in flies fed a HFD or CD (n = 10). Enterocytes of control animals (K) and of HFD treated animals (L) stained with DAPI. (M) Quantitative evaluation of the numbers of enterocytes (n = 10–11). CD = control diet, HFD = high-fat diet, ISC = intestinal stem cell, EB = enteroblast, EC = enterocyte, EMC = enteroendocrine mother cell, EEC = enteroendocrine cell, AM = anterior midgut, *p<0.05, **p<0.01, ***p<0.001.

In principle, there are two possibilities to explain the high numbers of esg^+^ cells, 1) the ISCs show an increased proliferation rate and 2) more esg^+^ cells are observed because their progression to enterocytes (EC) and enteroendocrine cells (EEC) is blocked (Fig 2A). To decide which of these two options applies in the case of HFD-induced increase of esg^+^ cells, we performed cell tracing studies using the ReDMM approach [30] (Fig 2B–2G). After induction, ISCs, EBs or enteroendocrine mother cells (EMCs) are simultaneously labeled with mCD8::GFP (GFP) and H2B::RFP (RFP), whereas newly generated cells (ECs and EECs) are exclusively marked by H2B::RFP. Thus, cells showing both fluorescent labels are ISCs, EBs or EMCs, whereas those that exhibit exclusively the red fluorescence are ECs or EECs (Fig 2A). After 3d of HFD (Fig 2B–2D) the number of cells showing both fluorescent labels was increased about twofold (Fig 2B–2D), whereas the number of cells only showing red fluorescence was not altered significantly (Fig 2B, 2C and 2G). After seven days of HFD no statistically significant difference could be observed in GFP and RFP expressing cells (representing ISCs, EBs and EMCs), but for only those cells that show RFP fluorescence only an app. twofold increase could be observed between control animals and those subjected to HFD (Fig 2E–2G). Moreover, we evaluated the effects of a HFD on the intestinal cellular composition and counted the numbers of EECs, which are direct descendants of EBs or EMCs (Fig 2A and 2H–2J). In comparison to intestines from control animals (Fig 2H), the anti-prospero staining revealed an increase in EEC cell numbers (Fig 2I). A quantitative evaluation of the number of EEC cells in a time-dependent manner revealed an increase in this cell group over time (Fig 2J). This increase started at day 3, peaked at day 7, and was sustained for more than 2 weeks in comparison with flies fed a CD. Thus, this time course of the increase of EEC cell numbers in response to HFD correlates nicely with the time dependency of EEC development, implying that the increased number of EBs did not result from a developmental arrest [31]. We also counted the numbers of ECs at day 7 following a HFD (Fig 2K–2M) and found no significant differences in the numbers of ECs in control (Fig 2K) compared to HFD animals (Fig 2L and 2M).

**Fig 3:**
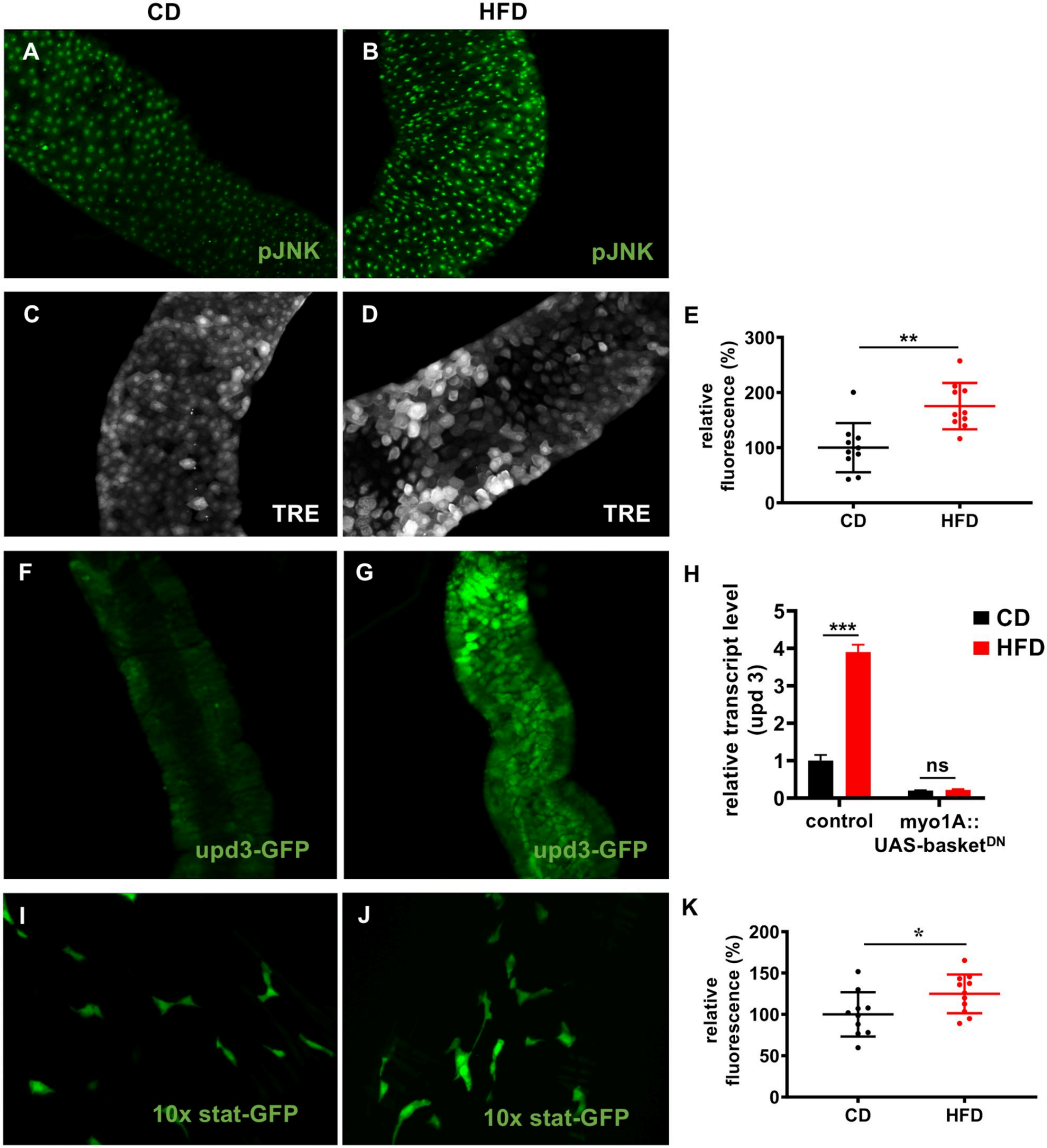
A HFD induces upd3 expression via JNK signaling. (A–B) Staining with an anti-pJNK antibody in intestines of flies fed a CD (A) or a HFD for 3 days (B). (C-D) Representative images of intestines of a JNK reporter line (4XTRE-DsRed) fed a CD (C) or HFD (D). (E) Fluorescence quantification in the anterior midgut region of the JNK reporter line upon control dieting or high-fat dieting (n = 8–10). (F-G) An upd3-GFP in vivo reporter was used to monitor upd3 expression in the intestines of flies fed a CD (F) or a HFD for 3 days (G). Representative images of the anterior midgut R2 region of the intestines are shown. (H) qRT-PCR analysis of upd3 expression in intestines of flies that expressed a dominant-negative form of basket in enterocytes (myo1A-Gal4::UAS-basket^DN^) or the control (w^1118^::UAS-basket^DN^) and fed a CD or a HFD for 3 days (n = 5). (I, J) Images of intestines isolated from a STAT-GFP in vivo reporter strain fed a CD (I) or HFD (J). (K) Quantification of fluorescence in the STAT-GFP reporter strain fed a CD or HFD for 3 days (n = 11). CD = control diet, HFD = high-fat diet, *p<0.05, **p<0.01, ***p<0.001.

JNK signaling is a major stress-sensitive signaling pathway in the intestines. Therefore, we tested if a HFD affects activation of JNK signaling (Fig 3). In comparison with flies fed a CD (Fig 3A), the level of phosphorylated JNK (pJNK), which corresponds to activated JNK, was increased in flies fed a HFD (Fig 3B). Furthermore, using a JNK reporter line [32] (4xTRE-dsRed), we showed that the level of fluorescence in the anterior midgut was significantly higher in flies fed a HFD than in flies fed a CD (Fig 3C–3E). The cytokine upd3, which is directly linked to stem cell activation, is a major target of JNK signaling in ECs [33]. Thus, we investigated the effects of a HFD on expression of upd3. Using a reporter line (upd3-Gal4::UAS-GFP), we showed that, in comparison with flies fed a CD (Fig 3F), expression of *upd3* in ECs was increased in flies fed a HFD (Fig 3G). qRT-PCR revealed that the transcript level of *upd3* was significantly increased in flies fed a HFD (Fig 3H). To determine whether HFD-induced JNK activation is causally linked with upd3 activation, we investigated if inhibition of JNK signaling via ectopic overexpression of a dominant-negative basket allele (*bsk*^*DN*^) in ECs affected *upd3* expression. HFD feeding did not increase *upd3* expression in flies expressing *bsk*^*DN*^ in ECs (Fig 3H), implying that activation of JNK upon HFD feeding is responsible for the increase in local *upd3* production and consequently stem cell activation. It is well established that upd3 produced by ECs induces proliferation of ISCs via activation of JAK/STAT signaling. Therefore, we used a STAT reporter line, in which GFP expression indicates JAK/STAT pathway activation [34]. As expected, GFP was detected in cells with a stem cell-like appearance scattered throughout the intestines. Fluorescence was relatively weak in flies fed a CD (Fig 3I), but was far stronger in flies fed a HFD (Fig 3J). Semi-quantitative analysis revealed that the level of fluorescence was significantly increased in flies fed a HFD (Fig 3K).

**Fig 4:**
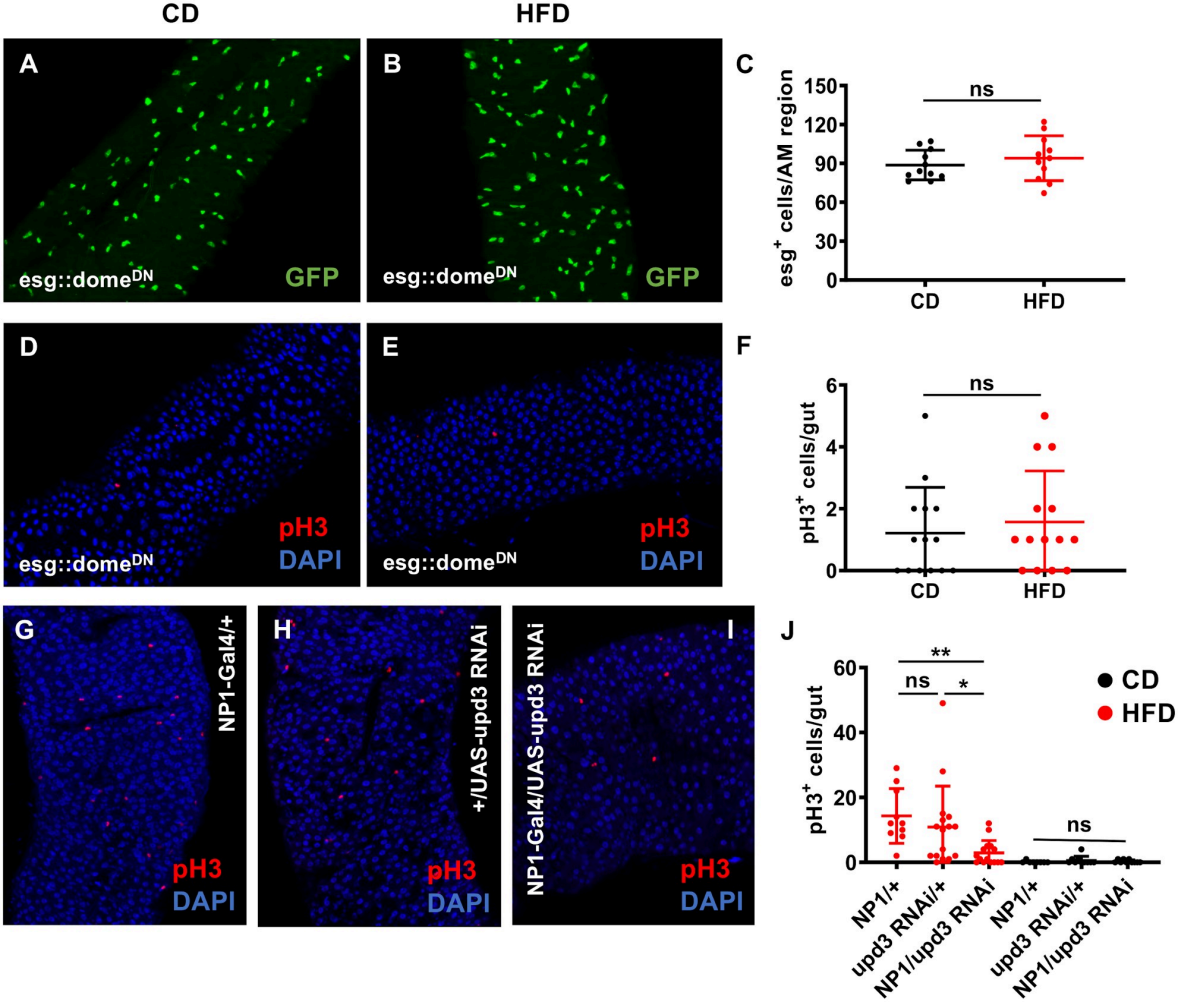
HFD-induced cell proliferation depends on upd3 expression in enterocytes and on JAK/STAT signaling in ISCs. (A–F) The esg-Gal4::UAS-dome^DN^ strain was used to block JAK/STAT signaling in ICSs. (A) Esg-Gal4::UAS-dome^DN^, UAS-gfp flies were subjected to control diet, while the same flies were fed with a HFD (B). (C) A quantitative analysis of these data was performed. The same type of experiments (as in A-C) was performed with the flies of the same genotype that were stained with anti-pH3 antisera (D-F). In order to study the effects of upd3 depletion in enterocytes, NP1::wt flies were subjected to HFD and stained with anti-pH3 (G), similarly as the second genetic control (wt::UAS-upd3-RNAi, H), and the experimental group (NP1-Gal4:: UAS-upd3-RNAi, I). The quantitative evaluation pH3^+^ cells of the corresponding genotypes subjected to a CD or HFD is shown in J. (n = 8–16). CD = control diet, HFD = high-fat diet, AM = anterior midgut,*p<0.05, **p<0.01.

To evaluate, if upd3 in ECs and STAT signaling in ISCs are indeed required for the observed HFD induced response, we used ectopic overexpression of a dominant negative isoform of the domeless receptor in ISC (and EBs) using the esg-Gal4 driver line (esg-Gal4XUAS-*dome*^DN^, Fig 4A–4F). In the intestines of those flies subjected to a control diet, a baseline number of esg^+^ cells was observed (Fig 4A), and HFD did not increase this number significantly (Fig 4B and 4C). This lack of response was also seen, if pH3^+^ cells were analyzed (Fig 4D–4F). The number of pH3^+^ cells in these animals (Fig 3D) was almost identical if compared with those subjected to a HFD (Fig 4E and 4F). To analyze the importance of ECs for HFD induced activation of stem cell division, we reduced the expression of the cytokine upd3 in these cells using RNAi. In control animals, HFD leads to an increased number of pH3^+^ cells in the gut. This can be observed for both genetic controls (Fig 4G, 4H and 4J). In contrast, the reduction of upd3 expression leads to a significantly reduced occurrence of pH3^+^ cells under these experimental conditions (Fig 4I and 4J). The low number of pH3^+^ cells under normal nutritional conditions is not affected by these interventions (Fig 4J).

**Fig 5:**
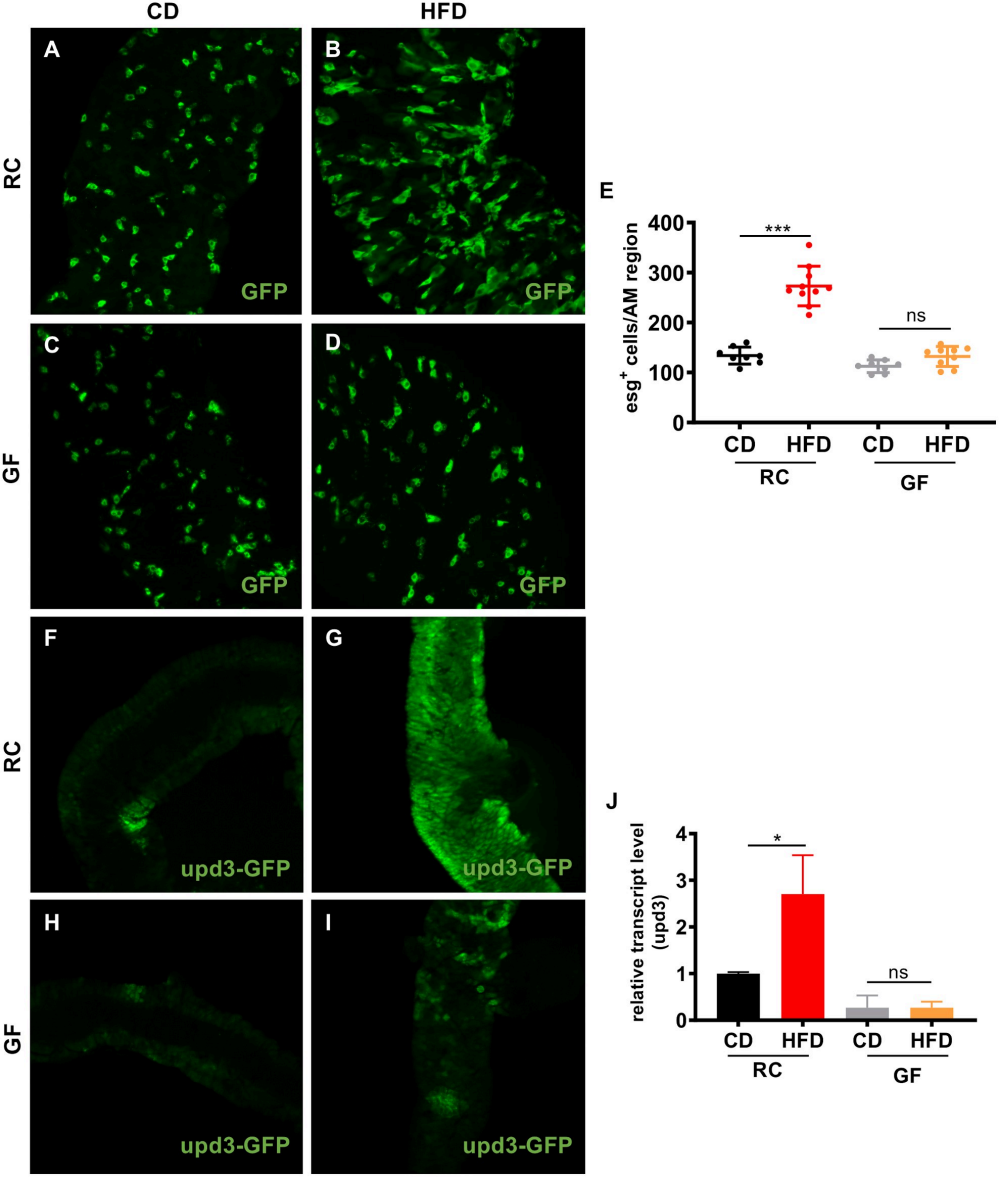
HFD-induced cell proliferation and upd3 expression are dependent on the intestinal microbiota. (A–D) The esg-Gal4::UAS-GFP strain, in which ISCs and EBs were labeled, was examined. Flies reconstituted with a normal microbiota were fed a CD (A) or HFD (B). GF flies were fed a CD (C) or HFD (D). Representative images of the anterior midgut region are shown. (E) Number of esg^+^ cells in the anterior midgut region of the intestines (n = 10). (F–I) An upd3-GFP reporter was used to examine microbiota-associated modulation of upd3 expression. Flies reconstituted with a normal microbiota were fed a CD (F) or HFD (G). GF flies were fed a CD (H) or HFD (I). Representative images of the anterior midgut are shown. (J) qRT-PCR analysis of upd3 expression in GF and RC flies fed a CD or HFD (n = 5). CD = control diet, HFD = high-fat diet, GF = germ-free, RC = recolonized, ISC = intestinal stem cell, EB = enteroblast, AM = anterior midgut, *p<0.05, ***p<0.001.

Dietary interventions affect the indigenous microbiota. Therefore, we compared germ-free (GF) flies with those that had been reconstituted with a natural microbiota (Fig 5). The latter flies had been used in all previously described experiments. As mentioned earlier, a HFD induced stem cell proliferation, which could be visualized by labeling ISCs and EBs with GFP using esg-Gal4::UAS-GFP flies. In those flies reconstituted with a natural microbiota, there were fewer esg^+^ cells if they were fed a CD (Fig 5A) compared to flies fed a HFD (Fig 5B). In GF flies, animals on CD showed a comparable number of esg^+^ cells as observed in animals reconstructed with a microbiota (Fig 5C and 5E). However, HFD in these animals had no increasing effect on the number of esg^+^ cells (Fig 5D and 5E).

This lack of induction in GF flies was also observed using reporter strains that allowed visualization of *upd3* expression (Fig 5F–5J), indicating that GF flies lack the signal necessary to induce proliferation of ISCs upon HFD feeding. Whereas the upd3 signal was low in flies reconstituted with a native microbiota and fed a CD (Fig 5F), it was sustainably increased upon HFD feeding (Fig 5G). By contrast, the upd3 signal was very low in GF flies, regardless if they were fed a CD (Fig 5H) or a HFD (Fig 5I). qRT-PCR analyses revealed that the transcript level of *upd3* was significantly lower in GF flies fed a HFD or CD than in flies reconstituted with a functional microbiota (Fig 5J) and that no induction of expression could be observed in HFD treated animals.

**Fig 6:**
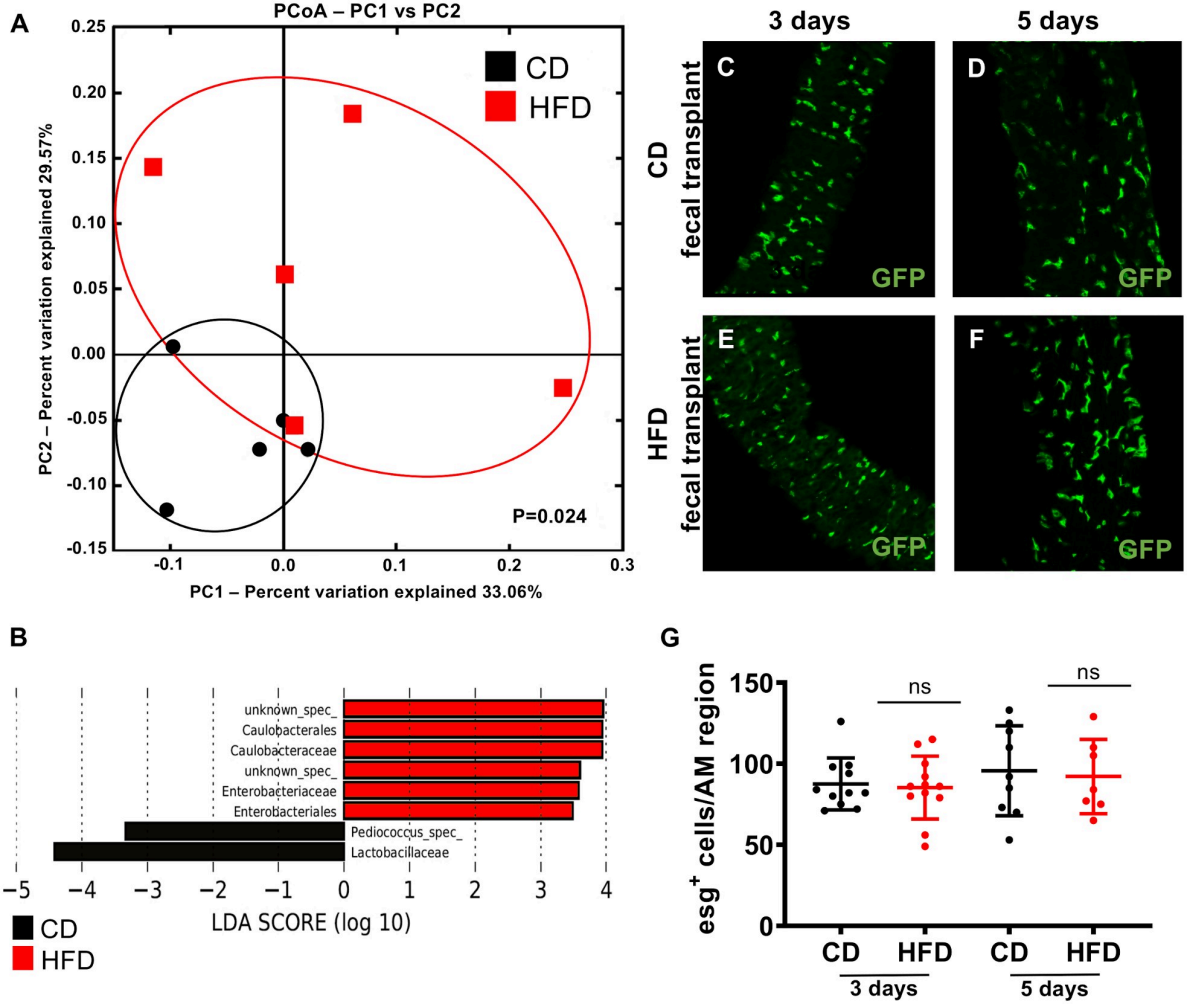
A HFD alters the microbial composition in w^1118^ flies. (A) Principal coordinate analysis showing significant alterations in the intestinal microbiota triggered by a CD or HFD. Each data point corresponds to one biological replicate. (B) Linear discriminant analysis effect size (LEfSe) to determine differentially enriched bacteria in the intestinal microbiota of flies fed a CD or HFD. (C–F) Fecal transplantation into the esg-Gal4::UAS-GFP reporter strain was performed to examine the effect of diet-induced changes in the microbial composition on intestinal cell proliferation. Flies were reconstituted with a microbiota derived from flies fed a CD (C–D) or HFD (E–F). Samples were analyzed after 3 days (C and E) or 5 days (D and F). (G) Quantitative analysis of the data presented in (C–F) (n = 8–12). CD = control diet, HFD = high-fat diet.

To assess the effects of HFD on the composition of the microbiota, we performed 16s rDNA-based analyses of microbial communities in control and HFD treated animals. A HFD significantly altered the composition of the microbial community in comparison with a CD, as illustrated by the separation in beta diversity by principal coordinate analysis based on unweighted UNIFRAC (Fig 6A). Linear discriminant analysis effect size (LEfSe) revealed that the orders Enterobacteriales and Caulobacterales were significantly enriched upon HFD feeding. On the other hand, flies fed a CD exhibited enrichment of species belonging to the family Lactobacillaceae, especially from the genus *Pediococcus* (Fig 6B). To determine whether these alterations in the microbial community were sufficient to induce stem cell activity, we performed a fecal transplantation assay (Fig 6C–6G). Specifically, we transferred the microbiota of flies fed a CD or HFD into GF flies expressing GFP under the control of the esg-driver. Transfer of the microbiota from flies fed a CD did not affect the number of esg^+^ cells after 3 days (Fig 6C) or 5 days (Fig 6D). In addition, transfer of the microbiota from flies fed a HFD did not increase the number of these cells after 3 days (Fig 6E) or 5 days (Fig 6F). Quantitative analysis of esg^+^ cells revealed that neither transfer affected stem cell activity in the intestines (Fig 6G), indicating that alteration of the microbiota composition does not underlie the increase in stem cell activity observed upon HFD feeding.

**Fig 7:**
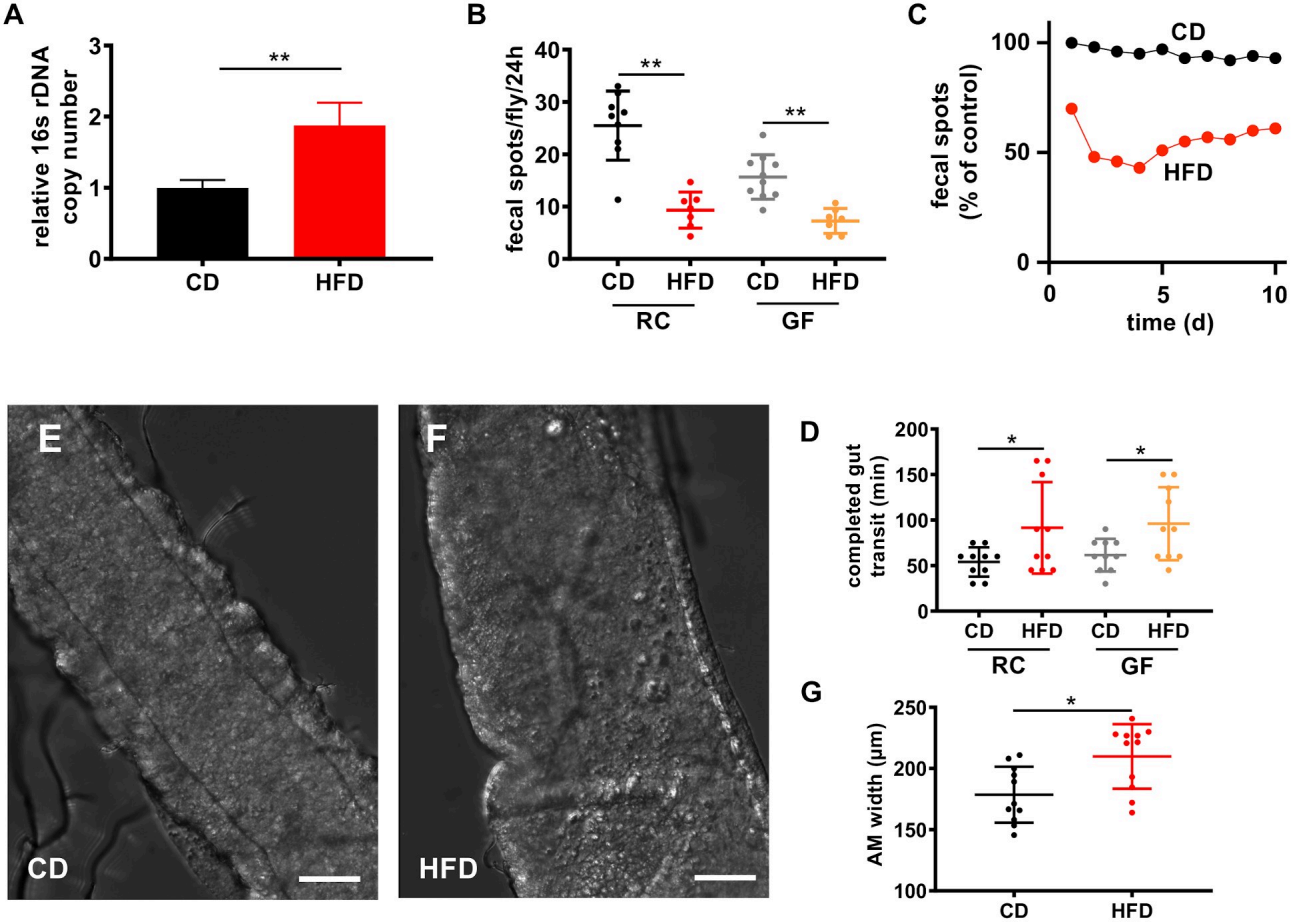
A HFD affects egestion and microbial abundance. (A) The intestinal bacterial load was analyzed by qRT-PCR using 16s universal primers. Intestines were dissected from flies fed a CD or HFD for 3 days (n = 5). (B) Analysis of fecal spot production over 24 h in flies with a reconstituted microbiota and GF flies fed a CD or HFD (n = 8–10). (C) Fecal spot analysis (compared to starting value (= 100%) under control conditions over time. (D) Gut transit time, defined as the time from food ingestion to egestion, was assessed by feeding flies blue food and determining the time until excretion of blue feces (n = 8–10). (E, F) Representative images of the anterior midgut region of flies fed a CD (E) or a HFD for 3 days (F, scale bar = 50 μm). (G) Quantification of the intestinal thickness of flies fed a control or a HFD (n = 11). CD = control diet, HFD = high-fat diet, GF = germ-free, RC = recolonized, *p<0.05, **p<0.01.

Besides the composition of the microbiota, HFD also influences the amount of bacteria in the intestine. The bacterial load in the intestines was ~2-fold higher in flies fed a HFD than in flies fed a CD (Fig 7A). Furthermore, a HFD induced mild constipation and consequently the number of fecal spots deposited over 24 h was substantially reduced by ~60–70% (Fig 7B). In addition, the number of deposited fecal spots was almost 50% lower in GF flies than in flies reconstituted with a functional microbiota. A HFD significantly reduced fecal spot production both in flies reconstituted with a functional microbiota and GF flies. This response was seen for a prolonged period of up to 10 days (Fig 7C). The gut transit time was shortest in flies reconstituted with a functional microbiota and fed a CD, but was increased in flies fed a HFD (Fig 7D). This effect was more pronounced in GF flies. The intestinal diameter was larger in flies fed a HFD than in flies fed a CD (Fig 7E–7G), which may reflect the increased bacterial mass in the intestines.

**Fig 8:**
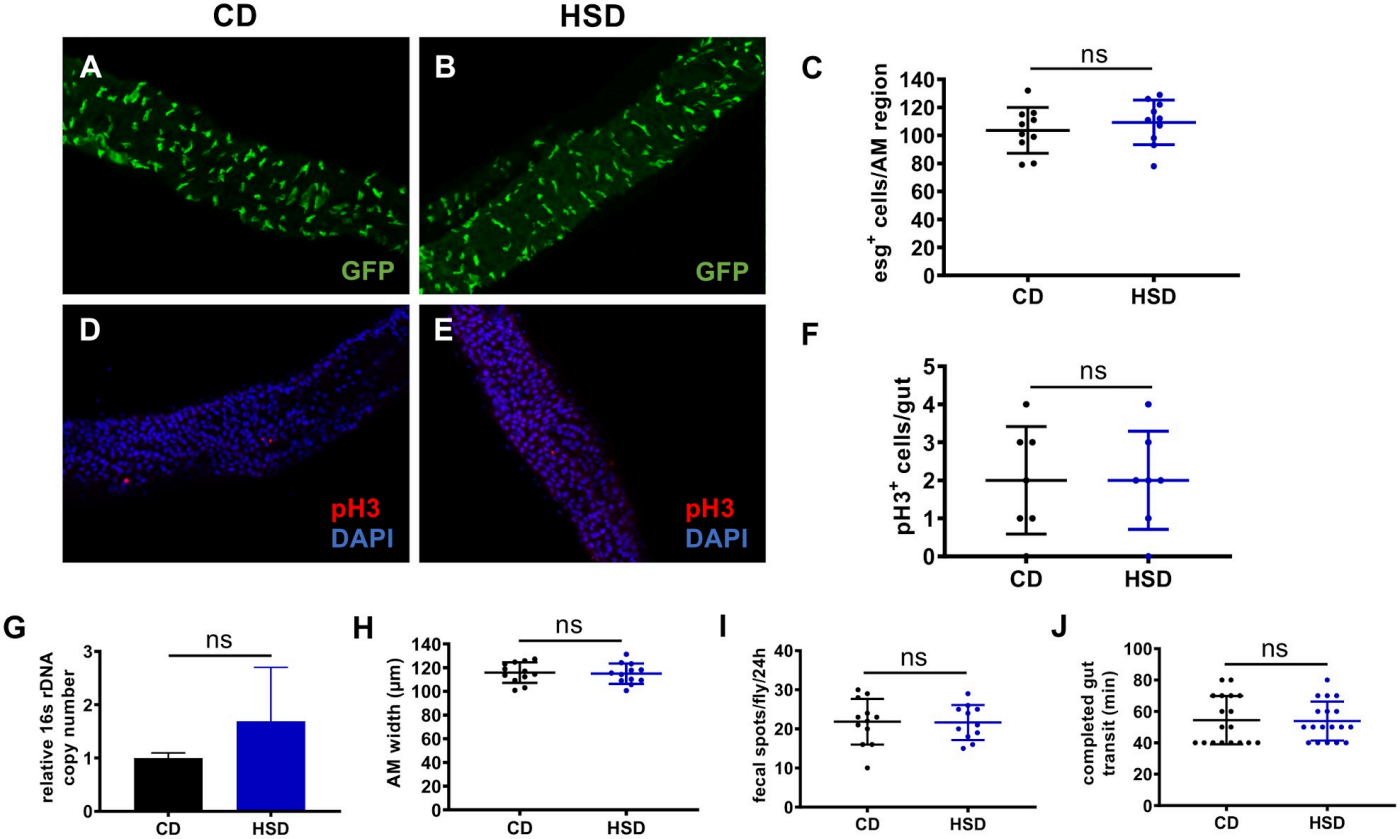
A high-sugar diet does not affect ISC proliferation, egestion or microbial abundance. (A-B) Representative images of the anterior midgut region of flies with GFP-labeled ISCs and EBs (esg-Gal4::UAS-GFP) and fed a CD (A) or a HSD (B) for 3 days. (C) Quantitative evaluation of esg^+^ cells in the anterior midgut region of CD or HSD fed flies (n = 10). (D-E) Representative images of anti-pH3 staining in intestines of flies fed a CD (D) or HSD (E). (I) Quantitative analysis of the number of pH3^+^ cells in whole intestines of HSD fed flies in comparison to CD fed flies (n = 7). The intestinal bacterial load of flies fed a CD or HSD for 3 days was analyzed by qRT-PCR using 16s universal primers (n = 5). (H) Quantification of the intestinal thickness of CD or HSD fed flies (n = 12). (I) Number of fecal spots and gut transit time (J) of flies fed a CD or HSD (n = 12–19). CD = control diet, HSD = high-sugar diet, AM = anterior midgut.

In order to test the hypothesis that HFD-induced stem cell proliferation is based on an increased energy supply coming along with the high-fat content, we employed an isocaloric diet where the extra calories are supplied by sugar rather than be fat (Fig 8). Subjecting esg-Gal4/UAS-GFP flies that express GFP in ISCs and EBs to the high-sugar diet (HSD) had no effect on the number of esg^+^ cells (Fig 8A–8C). Furthermore, we quantified the numbers of pH3^+^ cells in controls and HSD treated animals (Fig 8D–8F). In the intestines of control and HSD-treated flies, the bacterial load was quantified, revealing no difference between both treatment groups (Fig 8G). Consequently, we also quantified the intestinal width, which reflects the degree of intestinal filling, showed also no difference between animals on control diet and those subjected to HSD (Fig 8H). Finally, we quantified fecal spots per time (Fig 8I) and gut transit times (Fig 8J). For both parameters, no difference could be identified.

## Discussion

High-fat or lipid-rich diets are among the most important factors responsible for the development of metabolic diseases such as obesity and type 2 diabetes. The current study used the fruit fly *Drosophila melanogaster* as a simple model and focused on the effects of a HFD on the structure and functionality of the intestines, which is the first organ exposed to any dietary intervention. We found that a HFD induced a substantial increase in stem cell activity in the intestines, leading to altered cell numbers and changes in the cellular composition of this organ. The experiments in this study were conducted largely with palm fat, which is characterized by being particularly rich in triglycerides with saturated fatty acids. We first checked whether this lipid group is actually responsible for the observed effects by testing pure triglycerides with identical saturated fatty acids. Regardless of the chain length, these triglycerides can induce the fully developed phenotype, which clearly proves that this lipid population is responsible for the observed effect. In addition, we were able to verify that the increased occurrence of GFP-positive cells, representing ISCs and EBs, is not due to a developmental arrest, but to an actually occurring increased proliferation of stem cells. This was shown with two completely independent approaches. Firstly, we have shown by ReDMM lineage tracing [30] that terminally differentiated cells are actually produced in higher numbers. Secondly, we were able to show that one of the terminally differentiated cell populations, the EECs, are found in significantly higher numbers in the gut after HFD. Both findings exclude a developmental arrest as the underlying reason for the observed increase in GFP-positive cells (ISCs and EBs) in response to a HFD.

In addition to describing this particularly interesting phenotype, we have also elucidated some of the physiological and molecular mechanisms underlying this interesting phenomenon. First of all, and probably most importantly, we were able to show that this phenotype depends entirely on the presence of a functional intestinal microbiota. A HFD did not increase stem cell activity in germ free (GF) flies. Furthermore, we were able to elucidate essential aspects of the signalling pathway in enterocytes and the signalling pathway in ISCs, which are responsible for the expression of the phenotype. In ECs, which are in direct contact with all diets, JNK as well as parts of the JAK/STAT signaling pathways are essential for the HFD induced stem cell proliferation. We assume that JNK signaling transduces this microbiota-dependent signal in ECs, leading to production and release of the cytokine upd3, which in turn, activates JAK/STAT signaling in ISCs. We were able to show that both components of the JAK/STAT pathway in the gut, cytokine production by ECs and signal reception by ISCs are essential components for the transduction of a HFD into increased cell proliferation.

Different effects on the intestinal structure and intestinal functionality have been described in a variety of systems. These are on the one hand different lipid classes and on the other hand different animal models. In *Drosophila*, diets containing very high cholesterol concentrations significantly increased the abundance of EECs. This is due to a direct interaction with Notch signaling, which leads to preferential production of enteroendocrine cells [7]. Moreover, reduction of the lipid content decreases the proliferation of enteroendocrine tumors in this model [7]. Other lipid-rich diets induce comparable effects in mice, which are mainly characterized by increased cell proliferation in the intestines [35–37]. Beyaz and colleagues [9, 10] reported that a HFD induces stemness of ISCs, which is accompanied by decoupling of stem cell activity from its niche. This effect is opposite to the augmentation of stem cell function observed in response to dietary restriction in the intestines, which is dependent on increased integration into the stem cell niche rather than decoupling [4]. Comparable with the effects of cholesterol in the intestines of *Drosophila*, the effects of [38]a HFD in these experiments can be traced back to the direct interactions of particular fatty acids with ISCs [10].

As pointed out, these previously reported effects of HFDs on stemness are due to direct interactions between specific compounds in these diets (cholesterol or fatty acids) and intestinal cells. This is in contrast with the mechanism by which a HFD composed of triglycerides with saturated fatty acids promotes stemness in our system, which is indirect because it strictly requires the microbiota. Moreover, we choose a source of the HFD that is composed almost entirely of triglycerides with mostly saturated fatty acids and not of cholesterol. HFDs have been associated with dysbiotic microbiota, which has been causally linked with various pathogenic states [38]. The microbiota is also dysbiotic during aging [27, 28]. Most previous studies focused on the microbiota composition and reported that a shift toward specific bacterial colonizers correlates with the potential for disease development in the host. This appears to be irrelevant in our *Drosophila* system, because fecal transfer experiments failed to recapitulate the enhanced stemness phenotype. This is nevertheless not a final proof that the dysbiotic microbiota in HFD treated animals is not the driving factor for stem cell proliferation. Fecal transplantation experiments, especially those performed in *Drosophila*, can only define the starting microbial population that colonizes the intestine, but it does not allow control over time of the composition of this community. Instead, an increased abundance of bacterial colonizers appears to be more relevant. Two factors likely contribute to the increased microbial abundance in response to a HFD: 1) the high-energy content, which facilitates bacterial growth within the intestines, and 2) the increased gut transit time, which reduces fecal output and thus loss of bacteria. By testing an isocaloric diet in which the fat fraction was replaced by a carbohydrate fraction, we were able to show that the increased energy level plays no role. On the other hand, the gut transit time was increased and fecal output was reduced in flies fed a HFD, and these changes might underlie the HFD-induced increase in microbial abundance. Apparently, this response is chronic, which suggests that the underlying effect is also long-lasting. An increased gut transit time upon HFD feeding is also observed in mice and humans [39–41], indicating that it is a general response to this nutritional intervention and that a HFD is usually associated with an increased bacterial abundance. One consequence of the increased microbial abundance is elevated pressure within the intestinal lumen, leading to mechanical stress on the intestinal structure. This provides another scenario to explain how a HFD increases the stemness of ISCs; specifically, increased pressure induces an increase in stem cell activity in the intestines [42]. The piezo channel in precursors of enteroendocrine cells responds to mechanical stress, and this leads to production of enteroendocrine cells, which is consistent with the results of this study. We also showed that JNK signaling in enterocytes is essential for a suitable cellular response to such stress [43, 44]. This sentinel function of enterocytes is highly relevant because these cells are confronted with a plethora of stress signals and must signal about their state to enable their replacement if necessary [45, 46]. It has to be kept in mind that our hypothesis that higher microbial load is responsible for the enhanced stem cell activity can not be tested in a straight-forward experimental design. The experimental manipulation of the bacterial load is not possible without interference of other confounders in *Drosophila*.

In addition to the effects of a HFD on stem cell activity in the intestines, we also found that this dietary intervention induces long-lasting modifications of the intestinal hormonal architecture. A HFD increased the number of enteroendocrine cells for a considerable duration. This observation is consistent with previous reports that a HFD has multiple effects on intestinal function and homeostasis [47] and changes the expression and release of gut hormones [48]. These hormones play a central role in metabolic control and regulate various aspects of intestinal function [49]. We cannot rule out the possibility that feeding of a HFD for even a relatively short duration has long-lasting effects on intestinal homeostasis. In mammals, the substantial and long-lasting effects of short episodes of HFD feeding (e.g., only 3 days) attenuate major lipid-sensing systems in the gut [50]. HFDs usually reduce appetite via hormonal circuitry [51] to effectively prevent overnutrition [52]. Whereas almost nothing is known about the effects of HFDs on release of gut hormones in *Drosophila*, a plethora of studies have reported such effects in various mammals [53, 54]. Chronic exposure to HFDs increases release of the major gut hormone cholecystokinin in rats [55]. Similar effects have been reported for other gut hormones such as glucagon-like peptide 1 [56]; however, the underlying mechanism remains to be elucidated. Few interventions have been shown to change the number of enteroendocrine cells in the intestines [57]. Such a change would not only modify hormonal and metabolic homeostasis, but may also alter the stem cell niche because enteroendocrine cells are highly relevant for maintenance of this important niche [58].

Taken together, our results clearly show that a HFD, especially triglycerides with saturated fatty acids, elicits major effects on intestinal structure and function even in the simple model organism *D*. *melanogaster*. These effects include transient activation of stem cell activity and long-lasting changes to the cellular architecture in the intestines. Moreover, these effects are completely dependent on the microbiota and involve the stress-sensing JNK as well as the JAK/STAT signaling pathways in ECs and in stem cells.

## Material and methods

### Fly lines

The following fly strains were used in this study: *w*^*1118*^ (Bloomington Stock Center), *esg-GFP*, *UAS-dome*^*DN*^ (gifts from N. Perrimon, Harvard University, USA), *UAS-Luc* (gift from M. Markstein, University of Massachusetts, USA), *su(H)GBE-Gal4* (gift from S. Hou, Frederick, USA), *UAS-basket*^*DN*^ (Bloomington Stock Center 44801), *UAS-upd3-RNAi* (Bloomington Stock Center 32859), *10XSTAT*::*GFP* (gift from E. Bach, New York, USA), *20xUAS-IVS-mCD8*::*GFP* (Bloomington Stock Center 32194), *NP1-Gal4* (gift from D. Ferrandon, Strasbourg University, Strasbourg, France), 4XTRE-DsRed (Bloomington Stock Center 59011) and ReDDM flies (gift from M. Dominguez, Alicante, Spain).

### Fly food and husbandry

All flies were raised in vials on sterile standard cornmeal medium containing 5% inactivated yeast (BD Bacto yeast extract), 8.6% cornmeal (Mühle Schlingemann, Waltrop, Germany), 5% glucose (Roth, Karlsruhe, Germany), and 1% agar-agar (Roth) in an incubator at 20°C and 65% humidity. At 3–5 days after hatching, adult flies were transferred to fresh sterile standard cornmeal medium or high-fat medium. High-fat medium was exactly the same as standard cornmeal medium except that it contained 20% (w/v) food-grade palm fat (Palmin). ReDDM flies were raised at 18 °C to prevent induction of GFP and RFP expression. At 3–5 after hatching, adult flies were transferred to standard cornmeal medium or high-fat medium at 29°C to activate GFP and RFP expression. The triglycerides Tripalmitin, Tristearin or Tricaprin (TCI Chemicals) were added to the standard cornmeal medium at a concentration of 20% (w/v). The high-sugar medium contained 42% glucose, which corresponds to the caloric value of the high-fat medium (7400 kJoule/l).

### Axenic flies

Flies were allowed to lay eggs on apple juice agar plates containing 2% agar-agar (Roth 5210.2) and 50% apple juice (Rewe Bio apple juice) for about 12 h at 25°C to prevent the presence of larvae. Eggs were collected by rinsing the apple juice agar plates with deionized water and transferred to net baskets. Thereafter, the eggs were bleached for 2 min with 6% sodium hypochlorite (Roth, Karlsruhe, Germany) and then washed with 70% ethanol (Roth) and double autoclaved water under sterile conditions. Bleached embryos were placed on sterile standard cornmeal medium. The lack of bacteria in emerged adults was tested by PCR using universal primers targeting bacterial 16S rDNA.

### Reconstitution of bacteria

A mixture of the following bacterial species, which were cultured as previously described [59], was used to reconstitute the natural microbiota: *Acetobacter pomorum* (OD_600_ = 0.7), *Lactobacillus brevis*^*EW*^ (OD_600_ = 8), *Lactobacilles plantarum*^*WJL*^ (OD_600_ = 6), *Enterococcus faecalis* (OD_600_ = 0.8), and *Commensalibacter intestini*^*A911T*^ (OD_600_ = 1.5) (kindly provided by Carlos Ribeiro, Lisbon, Portugal). To prepare the stock solution for recolonization, the following volumes of each liquid culture were combined in a 15 ml falcon tube: 2 ml of *A*. *pomorum*, 0.02 ml of *L*. *brevis*^*EW*^, 0.25 ml of *L*. *plantarum*^*WJL*^, 2 ml of *E*. *faecalis*, and 1 ml of *C*. *intestini*^*A911T*^. The bacterial mixture was centrifuged three times at 3000 rpm for 15 min and repeatedly resuspended in sterile phosphate-buffered saline (PBS). Finally, the mixture was centrifuged at 3000 rpm for 15 min and resuspended in 25% glycerol prepared in sterile PBS. Aliquots of 500 μl were stored at -20°C. A volume of 50 μl of the bacterial mixture was added to the surface of control or high-fat media and allowed to settle for 1 h at room temperature under sterile conditions. Thereafter, 3–5-day-old GF flies were transferred to the corresponding media.

### Fecal transplantation

Reconstituted *esg*-GFP flies aged 3–5 days were cultured on high-fat or control media for 7 days Subsequently, five flies were drowned in 70% EtOh to remove bacteria from the body surface and homogenized in 300 μl of MRS medium (BD Difco, Thermo Scientific, Braunschweig, Germany) using a Bead Ruptor 24 (BioLab Products, Bebensee, Germany). Fly debris was removed by centrifugation, the supernatant was mixed with 5% sucrose, and 200 μl of the mixture was applied to sterile filter paper. GF *esg*-GFP flies were allowed to feed on the filter paper for 24 h and then transferred to control media for 5 or 7 days. GFP^+^ cells in the anterior midgut region were counted.

### Body fat quantification

Total body triacylglycerols in flies were measured using a coupled colorimetric assay as described previously [18, 60]. Groups of five females were weighed and homogenized in 1 ml of 0.05% Tween-20 using a Bead Ruptor 24 (BioLab Products, Bebensee, Germany). Homogenates were heat-inactivated at 70°C for 5 min, centrifuged, and incubated with triglyceride solution (Thermo Fisher Scientific, Braunschweig, Germany) at 37°C for 30 min. A standard curve was prepared using glyceryl trioleate. Absorbance at 562 nm was measured.

### Immunohistochemistry

Intestines were dissected in PBS, fixed in 4% paraformaldehyde for 1 h at room temperature, washed three times with PBST (PBS containing 0.1% Triton X-100), and blocked in blocking buffer (PBST containing 5% normal goat serum) for 1 h at room temperature. Thereafter, intestines were incubated with a primary antibody diluted in blocking buffer overnight at 4°C, washed three times, and then incubated with a secondary antibody diluted in PBST overnight at 4°C in darkness. Subsequently, intestines were washed three times with PBST and mounted on slides in Mowiol 40–88. Images were acquired using a fluorescence microscope equipped with Apotome (Carl Zeiss Image Axio Vision, Jena, Germany). The following antibodies were used: anti-prospero from mouse (1:50, Developmental Studies Hybridoma Bank, Iowa City, USA, MR1A), anti-GFP from mouse (1:300, Developmental Studies Hybridoma Bank, Iowa City, USA 8H11), anti-pJNK polyclonal from rabbit (Promega, Mannheim, Germany), Alexa Fluor 488-labeled goat anti-mouse IgG (1:300, Jackson ImmunoResearch, Dianova, Hamburg, Germany), and Alexa Fluor 555-conjugated goat anti-mouse IgG (1:300, Cell Signaling Technology, Frankfurt, Germany).

### Fluorescence-based quantification of the *in vivo* STAT-GFP reporter

Intestines of 10xSTAT::GFP flies were dissected after CD or HFD feeding for 3 days and immediately fixed in 4% paraformaldehyde and washed three times with 0.1% PBST. All images were stacked with a thickness of 2 μm and an exposure time of 60 ms. Fluorescence of all cells in the field of view was measured using ImageJ. Corrected total cell fluorescence (CTCF) was calculated as follows: CTCF = integrated density–(area of selected cell × mean fluorescence of background readings).

### Luciferase assay

The luciferase assay was performed as previously described [61] with minor modifications. The intestines of five adult flies per replicate were collected in 150 μl of Glo Lysis Buffer (Promega, Mannheim, Germany, #E2661) and homogenized using a bead mill homogenizer (BioLab Products, Bebensee, Germany) for 2 min at 3.25 m/s. The homogenate was transferred to a new reaction tube and stored at -20°C until further processing. For luciferase measurement, samples were thawed on ice and 50 μl was transferred to a white flat-bottom 96-well plate, with at least one empty well between treatments. Samples were mixed with the same amount of substrate provided by the One Glo Luciferase Assay System (Promega, Mannheim, Germany, #E6110) immediately before signal detection. Luciferase signals were detected using a Tecan plate reader (Tecan, Infinite M200 Pro, Männedorf, Switzerland). A defined control was included on each plate to normalize treatments across plates.

### Assessment of fecal output

A small piece of CD or HFD supplemented with Brilliant Blue FCF food dye (E133, Ruth, Bochum, Germany) was transferred to the bottom of a vial. A coverslip was placed in the middle of the vial to split it into two halves. Individual flies were trapped in one half, together with the piece of food, and the vial was sealed with a foam plug. Flies were incubated at 20°C for 24 h. Coverslips were scanned. The numbers of fecal spots were counted manually.

### Assessment of the intestinal transit time

Each well of a 24-well plate was loaded with a small piece of CD or HFD supplemented with Brilliant Blue FCF food dye. Individual flies were starved for 24 h, transferred to the wells, and monitored every 15 min for 3 h. The appearance of the first dyed fecal spot determined the time from food ingestion to egestion in each individual fly, which was referred to as the transit time.

### RNA extraction and qRT-PCR

Total RNA was extracted from the midgut of adult female flies that had been kept on standard cornmeal medium or high-fat medium. qRT-PCR was performed as described previously [62]. The following primers were used: *upd3* forward (5’-GAGAACACCTGCAATCTGAA-3’) and *upd3* reverse (5’-AGAGTCTTGGTGCTCACTGT-3’). The primers 8FM (5′-AGAGTTTGATCMTGGCTCAG-3′) and Bact515R (5′-TTACCGCGGCKGCTGGCAC-3′) were also used to quantify the bacterial load.

### 16S amplicon sequencing

DNA was isolated from intestines containing fecal material using a DNeasy Blood and Tissue Kit (Qiagen, Hilden, Germany) following the manufacturer’s protocol for purification of total DNA from animal tissues and for pretreatment of Gram-positive bacteria. Intestines of 10 individual flies were pooled per sample to generate sufficient material. Extracted DNA was eluted from the spin filter silica membrane with 100 μl of elution buffer and stored at -80°C.

16S profiling and MiSeq sequencing were performed as described previously [63, 64] with modifications. The V3-V4 region of the 16S gene was amplified using the dual barcoded primers 341F (GTGCCAGCMGCCGCGGTAA) and 806R (GGACTACHVGGGTWTCTAAT). Each primer contained additional sequences for a 12-base Golay barcode, an Illumina adaptor, and a linker sequence [65]. PCR was performed using Phusion Hot Start Flex 2× Master Mix (NEB, Frankfurt, Germany) in a GeneAmp PCR system 9700 (Applied Biosystems, Thermo Fisher Scientific, Frankfurt, Germany) and the following program: 98°C for 3 min, 30 cycles of 98°C for 20 s, 55°C for 30 s, and 72°C for 45 s, followed by 72°C for 10 min and then 4°C hold. PCR was checked by agarose gel electrophoresis. Normalization was performed using a SequalPrep Normalization Plate Kit (Thermo Fisher Scientific, Darmstadt, Germany) following the manufacturer’s instructions. Equal volumes of normalized amplicons were pooled and sequenced on an Illumina MiSeq (2 × 300 nt).

MiSeq sequence data were analyzed using MacQIIME v1.9.1 [66]. Briefly, all sequencing reads were trimmed to retain only nucleotides with a Phred quality score of at least 20, and then paired-end assembled and mapped onto the different samples using the barcode information. Rarefaction was performed at 34,000 reads per sample to normalize all samples against the minimum shared read count and to account for differential sequencing depth. Sequences were assigned to operational taxonomic units (OTUs) using UCLUST and the Greengenes reference database (gg_13_8 release) with 97% identity. Representative OTUs were picked and assigned to a taxonomy using UCLUST and the Greengenes database. Quality filtering was performed by removing chimeric sequences using ChimeraSlayer and by removing singletons and sequences that failed to align with PyNAST. The reference phylogenetic tree was constructed using FastTree 2. Relative abundance was calculated by dividing the number of reads for an OTU by the total number of sequences in the sample. Unweighted Unifrac beta diversity was calculated and visualized by generating principal coordinate plots. Differentially abundant taxa were assessed using the nonparametric t test. p values were adjusted for multiple testing using the FDR correction. LEfSe [67] was performed using an online tool available at http://huttenhower.sph.harvard.edu/galaxy. LDA denotes taxa based on their contribution to the overall observed differences between groups, i.e., taxa whose abundance was significantly higher in flies fed a HFD than in flies fed a CD.

### Statistical analysis

All statistical analyses were performed using Prism 6.0 (GraphPad Software, San Diego, USA). Lifespan data were analyzed by the log rank test (Mantel-Cox).
